# Genomic Abnormalities as Biomarkers and Therapeutic Targets in Acute Myeloid Leukemia

**DOI:** 10.3390/cancers13205055

**Published:** 2021-10-09

**Authors:** Sara Ribeiro, Anna M. Eiring, Jamshid S. Khorashad

**Affiliations:** 1 Centre for Molecular Pathology, Royal Marsden Hospital, Sutton SM2 5PT, UK; sara.ribeiro@icr.ac.uk; 2 Center of Emphasis in Cancer, Department of Molecular and Translational Medicine, Paul L. Foster School of Medicine, Texas Tech University Health Sciences Center at El Paso, El Paso, TX 79905, USA; anna.eiring@ttuhsc.edu; 3 Division of Molecular Pathology, Institute of Cancer Research, Sutton SM2 5PT, UK; 4 Centre for Haematology, Department of Immunology and Inflammation, Imperial College London, London W12 0NN, UK

**Keywords:** acute myeloid leukemia, targeted therapy, drug resistance

## Abstract

**Simple Summary:**

AML is a heterogenous malignancy with a variety of underlying genomic abnormalities. Some of the genetic aberrations in AML have led to the development of specific inhibitors which were approved by the Food and Drug Administration (FDA) and are currently used to treat eligible patients. In this review, we describe five gene mutations for which approved inhibitors have been developed, the response of AML patients to these inhibitors, and the known mechanism(s) of resistance. This review also highlights the significance of developing function-based screens for target discovery in the era of personalized medicine.

**Abstract:**

Acute myeloid leukemia (AML) is a highly heterogeneous malignancy characterized by the clonal expansion of myeloid stem and progenitor cells in the bone marrow, peripheral blood, and other tissues. AML results from the acquisition of gene mutations or chromosomal abnormalities that induce proliferation or block differentiation of hematopoietic progenitors. A combination of cytogenetic profiling and gene mutation analyses are essential for the proper diagnosis, classification, prognosis, and treatment of AML. In the present review, we provide a summary of genomic abnormalities in AML that have emerged as both markers of disease and therapeutic targets. We discuss the abnormalities of RARA, FLT3, BCL2, IDH1, and IDH2, their significance as therapeutic targets in AML, and how various mechanisms cause resistance to the currently FDA-approved inhibitors. We also discuss the limitations of current genomic approaches for producing a comprehensive picture of the activated signaling pathways at diagnosis or at relapse in AML patients, and how innovative technologies combining genomic and functional methods will improve the discovery of novel therapeutic targets in AML. The ultimate goal is to optimize a personalized medicine approach for AML patients and possibly those with other types of cancers.

## 1. Introduction

The introduction of imatinib as a tyrosine kinase inhibitor (TKI) targeting *BCR-ABL1* revolutionised the treatment of patients with chronic myeloid leukaemia (CML), paving the path for the development of other targeted inhibitors in various types of cancers [1]. The development of imatinib was based on the concept that targeting the driver of malignancy (the BCR-ABL1 oncoprotein) rather than the consequence (proliferation, the target of standard chemotherapies) should control the disease without damaging normal cells that do not express the driver mutation [2]. Before imatinib, CML was a malignancy associated with early death from progression to acute leukaemia or from side effects associated with bone marrow transplantation, which was the only curative option. However, nearly two decades of treating CML patients with imatinib has been a great success for this novel targeted therapy [3]. The success of managing CML using targeted inhibitors has continued with the development of second and third generation BCR-ABL1 TKIs, which help clinicians to manage emerging resistance to imatinib [3]. In some patients, imatinib is so effective at suppressing leukaemia that patients maintain their deep molecular response even after they discontinue therapy (Stop Imatinib trial) [4].

The promising data from targeting BCR-ABL1 in CML encouraged the exploration of novel inhibitors for other cancers with known oncogenic drivers [5]. However, to date, the development of targeted inhibitors for other malignancies has not proven as successful as imatinib, partly due to the presence of several genomic abnormalities contributing to the development of most other types of cancers. In acute myeloid leukaemia (AML), for instance, an average of 13 genetic abnormalities can be observed per patient, and the heterogeneity of the disease along with ambiguity of the main driver among the detected mutations may explain why the development of novel inhibitors for AML has not been as successful [6]. To date, specific inhibitors have now been developed and used in clinical practice for certain subtypes of AML for which there is a well-known oncogene at the centre of disease pathogenicity, such as promyelocytic leukemia/retinoic acid receptor-alpha (PML/RARα) and FMS-like tyrosine kinase 3-internal tandem duplication (*FLT3*-ITD), or AML patients with mutations in isocitrate dehydrogenase isozymes 1 or 2 (IDH1, IDH2) [7,8]. In fact, AML patients with PML/RARα are curable with a combination of arsenic trioxide (As_2_O_3_) and all-trans retinoic acid (ATRA), which have shown higher success rates compared with other AML subtypes [9]. Although there has been promising data regarding the usage of FLT3 inhibitors in combination with azacytidine for the management of *FLT3*-ITD+ in AML patients, these inhibitors have not improved survival to the same level as imatinib in managing CML or ATRA in managing acute promyelocytic leukaemia (APL).

Because of the observed heterogeneity, AML has been classified into different subtypes, which provide information regarding probable outcome and treatment response. The World Health Organization (WHO) classification system has classified AML into the following subtypes: *AML with recurrent genetic abnormalities*, which include subtypes with recurrent cytogenetics or molecular genetics abnormalities such as acute promyelocytic leukemia (APL) with *PML-RARA*, AML with mutated *NPM1*, AML with biallelic mutations of *CEBPA*, etc.; *AML with myelodysplasia-related changes*; *therapy-related myeloid neoplasms*; *AML not otherwise specified (NOS)*, which includes cases that do not fall into any of the other groups (such as pure erythroid leukemia), acute monoblastic/monocytic leukemia, etc.; *myeloid sarcoma* (also known as granulocytic sarcoma or chloroma) and *myeloid proliferations related to Down syndrome* [10]. Heterogeneity of AML is the main obstacle to developing specific inhibitors for clinical management. Thus, personalised medicine offers an ideal approach for successful management of this disease [11]. The principle of personalised medicine is to identify the main pathway(s) that are essential for survival of the leukaemia cells and to target them in a patient-specific manner. The identification of these pathways can be achieved through genomic techniques such as RNA sequencing or whole exome sequencing. However, without functional studies, genomic techniques only provide information on the genetic aberrations and lack the power to characterise the activated signalling pathways that are responsible for the disease phenotype. In this review, we discuss the underlying genomic abnormalities which make AML patients eligible for targeted therapy and also highlight additional alterations that drive resistance to targeted therapies. The cited AML studies are mainly from adult AML patients. Additionally, we will briefly discuss how functional genomics might be used for identification of potential therapeutic targets in a personalised medicine approach for the clinical management of AML patients.

## 2. PML-RARA

**Figure 1:**
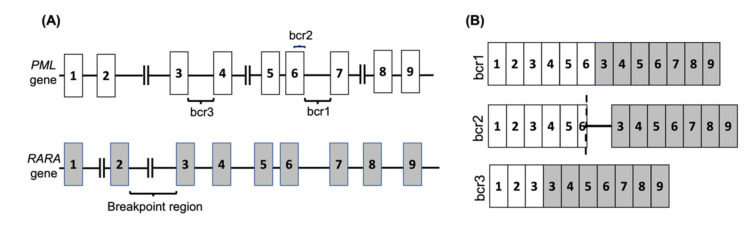
The *PML-RARA* fusion gene in APL. (**A**) The common breakpoint regions on the *PML* gene are shown, with the most common break occurring between exons 6 and 7 (bcr1) or exons 3 and 4 (bcr3), whereas the *RARA* gene breakpoint can occur anywhere within the 15kb intronic region between exons 2 and 3. In 5% of PML cases the breakage occurs in exon 6 (bcr2). (**B**) The common *PML-RARA* fusion mRNAs are shown. The brc2 variant mRNA contains a variable insertion from *RARA* intron 2 located between part of *PML* exon 6 (shown by dash) and *RARA* exon 3.

APL is one subtype of AML that is observed in nearly 12% of AML patients [12]. It is characterised by a translocation between the retinoic acid receptor-alpha (*RARA*) gene on chromosome 17 with the promyelocytic leukemia (*PML*) gene on chromosome 15, known as t(15,17)(q24, q12) in nearly 99% of APL cases. The *RARA* gene has also been shown to fuse with other partner genes in rare cases. The *PML-RARA* fusion is classified into three main categories depending on the breakage point on PML, and consequently the isoform of the resulting mRNA fusion (Figure 1 and Table 1).

The current standard induction treatment for APL with ATRA and anthracycline, followed by at least two additional cycles of consolidation therapy, has been shown to lead to complete remission (CR) in 95% of APL patients. ATRA simultaneously activates the transcription of genes essential for myeloid differentiation while also inducing the degradation of the PML-RARα protein [7]. The other agent which was shown to be an effective therapy in APL is As_2_O_3_. The significance of As_2_O_3_ is its effectiveness even in patients who relapse following ATRA/chemotherapy, as up to 80% of such cases have been shown to achieve complete remission following As_2_O_3_ therapy [19,20,21]. As_2_O_3_ induces the production of reactive oxygen species (ROS), which in turn causes multimerization of PML-RARα through intermolecular disulphide crosslinks at the PML B1-domains; binding of As_2_O_3_ to the C-C motif in PML-B2 was shown to be crucial for multimerization. ROS leads to increased ubiquitin carrier protein 9 (UBC9) binding to the PML RING domain, which increases PML-RARα SUMOylation and the recruitment of ring finger protein 4 (RNF4). This ultimately leads to polyubiquitination of PML-RARα protein and its subsequent degradation by the ubiquitin-proteasome system [22,23,24,25]. Response to treatment is usually evaluated by measuring *PML-RARA* mRNA transcripts using quantitative reverse transcription polymerase chain reaction (qRT-PCR), which is expected to be negative following consolidation therapy. Failure to achieve a negative PCR result post-consolidation (confirmed by two tests with two-week intervals) is considered primary resistance and necessitates additional therapeutic measures.

**Figure 2:**
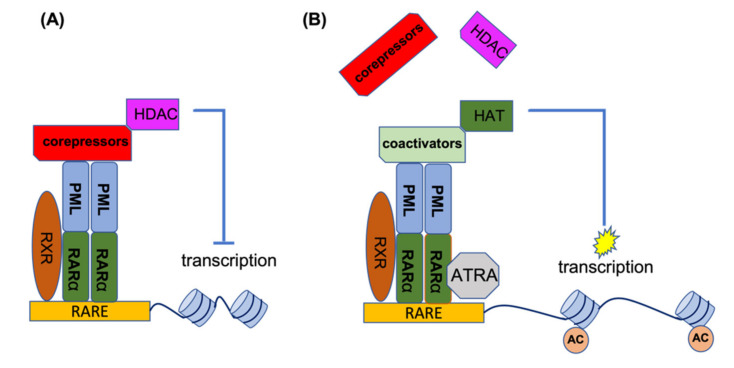
ATRA mechanism of action for the treatment of APL patients. (**A**) In the absence of ATRA, PML-RARα binds to RARE elements to recruit co-repressors and HDACs for suppression of gene transcription. (**B**) ATRA disrupts the co-repressor complex and provides an opportunity for the association of co-activator complexes with RARα, leading to histone acetylation and the activation of gene transcription. AC, acetylation of histones.

RARα is a transcription factor which, upon activation, binds to a specific element (RARE) in the promoter of its target genes. In the absence of the inducing ligand, RARα forms a heterodimer with the retinoic X receptor (RXR). This heterodimer recruits histone deacetylases (HDACs) to the promoter site, and by deacetylation of the histones, this leads to condensation of the RARα target genes. Under physiological conditions, retinoic acid (RA) can disrupt the RARα-RXR complex and release the HDAC, thereby recruiting activating factors to the promoter site, resulting in the transcriptional activation of target genes. In the event of PML-RARα formation, this complex binds to PML and other chimeric PML-RARα proteins (homo-and hetero-dimerization). In addition to HDACs, it also recruits DNA methylating enzymes such as DNA methyltransferases (DNMT3A and DNMT3B) to the promoter site, which methylates the promoter in addition to deacetylating the histones, leading to stronger target gene suppression. The co-repressors of the PML-RARα complex are not released by physiological levels of RA and are only released by ATRA. In the presence of ATRA, the co-repressor complex loses its ability to bind RARα, providing an opportunity to associate with the co-activator complex for the activation of gene transcription. Figure 2 shows the mechanism of action for ATRA treatment of *PML-RARA*-positive APL [26,27,28,29,30,31,32].

Primary clinical resistance, defined as failure to achieve complete remission on ATRA therapy, is very rare. However, secondary resistance has been observed in 10–30% of relapsed cases following complete remission [33]. A variety of mechanisms have been described for resistance to ATRA following relapse. However, for this review, we will only highlight the genomic alterations associated with resistance. As described above, the co-repressor complex is dissociated from PML-RARα in the presence of ATRA. However, mutations in the ligand binding domain (LBD) of the *RARA* gene can interrupt this interaction and are considered a mechanism of resistance to ATRA. Mutations in the *RARA* LBD in PML-RARα have been observed through clinical observation in resistant patients, and their resistance to ATRA has been validated through in vitro analysis of cell line models (Table 1) [13,34,35,36]. There are a few studies showing mutated genes at relapse following ATRA therapy. In one study, the APL cells with diagnostic mutations in *WT1* (D367_R369del, Q259*R458* K400* H465N), activating mutations of MAP kinase pathway genes (*BRAF*, *KIT*, *PDGFRA*), and inactivating mutations in the genes regulating transcription or epigenetics (*NSD1*, *ASXL1*, *MED12*, *KDM6A*) were observed in relapsed cells at a higher frequency [37]. At relapse, mutations of these genes were detected along with additional mutations which were not present at diagnosis. These mutations were either acquired during the progression of the disease toward relapse, or were present at low levels at diagnosis as a subclone and selected during the course of therapy (Table 2). In some *FLT3*-*ITD*+ APL patients, it was observed that the presenting clone at relapse lost the *FLT3*-*ITD* mutations or other passenger mutations that were present at diagnosis, indicating the existence of a pre-leukemic *PML-RARA*-expressing clone that survived RA/chemotherapy and reinitiated APL [37]. The combination of ATRA and As_2_O_3_ has greatly reduced the rate of relapse and improved the lives of APL patients [38], and represents one of the most successful targeted cancer therapies available today, second in line only to TKI treatment in CML.

## 3. FLT3

**Figure 3:**
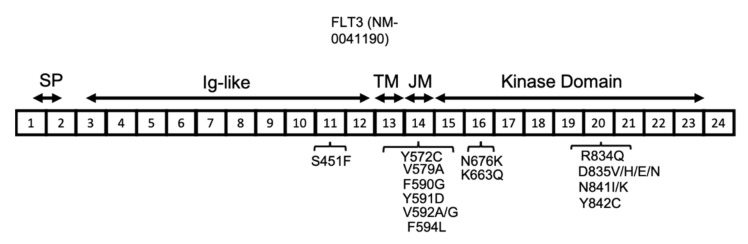
FLT3 protein structure in AML. FLT3 protein has a signal peptide (SP) at the NH2-terminal, followed by five extracellular Ig-like domains, a transmembrane (TM) domain, a juxta-membrane (JM), and a kinase domain. The JM and kinase domains are separated into two parts by a short region named the kinase insert (KI), which is not shown here. The location of the reported *FLT3* activating mutations are shown in association with their related exons (numbered 1–24).

The *FLT3* gene is located on chromosome 13 and has 24 exons, with translation starting from the middle of exon 1 and continuing through part of exon 24. The resulting human FLT3 protein is composed of 993 amino acids, with three main sections relating to the membrane, extracellular, and intracellular domains. The extracellular domain is composed of the signal peptide (encoded by exons 1 and 2 and is 26 amino acids long), which is cleaved off during processing, and also an extracellular immunoglobulin-like (Ig-like) domain (encoded by exons 3–12). The transmembrane domain (between amino acids 542 and 564) is encoded by exon 13, whereas the intracellular region, including the juxtamembrane and kinase domains (between amino acids 610 and 944, including the kinase insert of nearly 50 amino acids), is encoded by exons 14–23 (Figure 3) [40].

Human FLT3 is predominantly expressed in hematopoietic stem and progenitor cells, both myeloid and lymphoid, and has not been observed in mature cells, highlighting its significance for the early stages of hematopoietic development [40,41,42]. Several transcriptional factors, including HOXA9, MEIS1, PBX1, PBX3, CEBPα, MYB, and PAX5, regulate transcription of *FLT3* in both mouse and human cells [40]. The FLT3 protein goes through glycosylation in the endoplasmic reticulum and Golgi apparatus before it trafficks to the membrane [40,43,44]. Additional post-translational modifications known to affect FLT3 protein are phosphorylation and ubiquitination. Several tyrosine residues, including tyrosines 572, 589, 591, 599, 726, 768, 793, 842, 955, and 969 are phosphorylated during the ligand-induced activation of FLT3 [45,46,47]. In the monomeric membrane state, the FLT3 protein remains inactive, and this inactive state is maintained through interactions between the juxtamembrane and kinase domains [48]. This interaction blocks ATP from accessing the kinase domain site, preventing the phosphorylation and activation of tyrosine residues. However, the binding of the FLT3 ligand to FLT3 leads to dimerization and loss of interaction between the juxtamembrane and kinase domains, allowing ATP to access the tyrosine site for autophosphorylation. Tyrosine phosphorylation is the ultimate marker of FLT3 activation [48].

The majority of *FLT3* mutations have been observed in the juxtamembrane domain (JMD) and the adjacent tyrosine kinase domain (TKD) of the FLT3 protein, and are mainly comprised of insertion in-frame mutations, known as internal tandem duplication (ITD). Nearly 70% of ITD mutations have been reported to occur within the JMD, and the other 30% were identified mainly within the TKD [49,50]. Comparing the outcome of AML patients with FLT3-ITD suggests differences between the prognostic significance of JMD-ITD and TKD-ITD [51,52,53]. A recent analysis of a large population of patients from the RATIFY trial with *FLT3-ITD* mutations showed inferior outcomes for patients with TKD-ITD compared with JMD-ITD [53]. The ITD mutations disrupt the interactions between the JMD and the activation loop, leading to availability of ATP to the tyrosine residues and their phosphorylation, resulting in constitutive activation of the protein kinase domain [48,54].

Reports from several groups have shown that mutational load (measured by the ratio of mutant FLT3/non-mutant FLT3) has significant clinical relevance, with a higher ratio associating with poor prognosis [55,56,57]. The allelic ratio for *FLT3-ITD* refers to the number of ITD-mutated alleles compared with the number of wild-type alleles. AML patients with *FLT3-ITD*s have been shown to have poor outcomes by several large studies, particularly for patients with a high allelic ratio (≥0.5) [51,58,59]. Most of the *FLT3-ITD* mutations in the JMD have been reported in a tyrosine-rich region from codon 589 to 599 [40,49,60]. At a much lower frequency, ITD can also occur near the beginning of the TKD (Figure 3) [50]. ITD mutations account for 25% of the mutations in AML patients, with the size of the duplication ranging from 3 to 1236 bp. A large study of *FLT3-ITD*-positive AML patients showed simple duplication and/or insertions of unknown origin [61]. The same study showed some patients had 2-3 independent *FLT3-ITD*s in the region of exons 13–15. There is no general agreement on the prognostic significance of the *FLT3-ITD*, but the level of *FLT3-ITD* is commonly recognised as a prognostic factor for relapse in AML patients [57]. Recently, a novel mutation of a single amino acid residue in the *FLT3* JMD (Q575D) which activated the FLT3 kinase in a manner similar to ITD was reported in AML patients [62]. This is an important finding, as identification of more patients with such mutations will influence the current diagnostic methods for detection of *FLT3* mutations, and will recommend screening of the JMD by next generation sequencing in addition to fragment analysis [57]. In most of the AML patients with *FLT3-ITD* at diagnosis, the *FLT3-ITD* mutation is detected at a higher allelic burden at relapse [63]. However, other patterns have also been observed during the progression of the disease from diagnosis to relapse. Nearly 20% of patients with AML acquire either a newly detectable *FLT3-ITD* or *FLT3-TKD* mutation at relapse or lose the *FLT3* mutation from the time of diagnosis [64].

*FLT3* point mutations have also been reported among AML patients at a lower frequency compared with ITD (7% of AML patients) [40,61]. In contrast to ITD mutations, the prognosis of AML patients with *FLT3* point mutations is more favourable [65]. The most common codon affected by point mutation is D835, located near the activation loop, which can undergo a variety of mutations, including D835V, H, E, and N, all of which lead to FLT3 activation. The other less common activating point mutations in *FLT3* include S451F, Y572C, V579A, F590G, Y591D, V592A, V592G, F594L, K663Q, N676K, R834Q, N841I, N841K, and Y842C (Figure 3) [40,66,67,68,69,70,71,72]. The type of mutation influences the downstream signalling pathways that are activated. The FLT3-ITD protein is mainly located in the endoplasmic reticulum (ER), whereas the *FLT3* D835Y mutant is mainly located to the plasma membrane. The subcellular localization of FLT3 influences the downstream signalling pathways that are activated. For instance, the FLT3 protein, when located at the plasma membrane, stimulates proteins such as PI3K/AKT and mitogen-activated protein kinase (MAPK), whereas FLT3 with ITD mutations primarily activates the STAT5 signalling pathway [73,74].

Several inhibitors targeting FLT3 have been developed, and based on the underlying mechanism of FLT3 inhibition, they have been categorised into two classes [75]. The class I multi-kinase inhibitors include midostaurin, gilteritinib, and crenolanib, among which only midostaurin has been FDA and EMA (European Medicines Agencies)-approved for treatment of AML patients with activating *FLT3* mutations (in combination with cytarabine and daunorubicin) [40]. Gilteritinib, on the other hand, is approved for the treatment of relapse or for refractory AML patients [76]. The common feature of class I inhibitors is their binding to the “gatekeeper” domain adjacent to the activation loop or the ATP-binding domain in both active and inactive receptor conformations [75,77]. Class II inhibitors, such as sorafenib, quizartinib, and ponatinib, bind to the hydrophobic region of FLT3 (ATP-binding domain) in its inactive conformation and were developed to improve drug specificity [78].

Although the response to FLT3 inhibitors has been promising, the duration of response remains short, which is mainly due to the emergence of resistant clones [76,79,80,81]. The mechanism of evolving resistance to FLT3 inhibition has many features in common with the mechanisms of resistance to TKIs in other types of leukaemia. Usually at the time of resistance, there is a dominant clone, shown in several cases to be present as a minor clone at diagnosis, but selected and expanded at the expense of other clones that are suppressed during treatment. However, resistant clones might also develop during treatment as a result of acquiring new mutations [82,83]. Resistance may be due to point mutations that disrupt drug binding, or activation of alternative pro-survival signalling pathways in spite of FLT3 inhibition. According to a recent model proposed by Joshi et al., the mechanism of resistance to the FLT3 inhibitor gilteritinib changes during the evolution of resistance. Early resistance is mainly due to protective factors from the bone marrow microenvironment, metabolic adaptation, slower growth, and dependence on Aurora kinase B, while late resistance is due to mainly intrinsic mechanisms such as mutations in the RAS pathway and the continuation of altered metabolism. Available data from different studies suggests the involvement of various factors in the development of resistance, including both intrinsic and extrinsic factors [84].

Alterations of the *FLT3* gene, such as certain FLT3 kinase domain mutations, have been predicted to be resistant to some of the FLT3 inhibitors based on in vitro models, or have been observed in the resistant samples while they were absent from the diagnostic sample [85]. The mutations causing resistance to a drug of one class are highly likely to demonstrate resistance to other drugs of the same class due to common features of drug binding, but may remain sensitive to the drugs from the other class due to the difference in the binding site [86]. Class I FLT3 inhibitors are less specific for FLT3 and can bind the active and inactive conformation of the FLT3 protein. This explains the low frequency of *FLT3* mutations as a mechanism of resistance to this class of FLT3 inhibitors. This is in contrast with the pattern of resistance observed in the class II FLT3 inhibitor quizartinib, which has low inhibitory activity against *FLT3-TKD* mutations at therapeutic doses [78,87]. Mutations of the *FLT3-TKD* D835 or I836 amino acid were shown to be the main *FLT3* mutations associated with resistance to class II FLT3 inhibitors [88]. The available data suggests that nearly one third of patients who developed resistance to FLT3 inhibition have these mutations in *FLT3* at the time of relapse [85,88,89]. A recent investigation of 67 *FLT3-ITD*+ AML patients who were treated with class I or II inhibitors demonstrated that more than half of the patients had detectable mutations at relapse, from which 26% had mutations in the TKD of *FLT3* (D835). Interestingly, mutations of the *FLT3* TKD were more common in those treated with class II inhibitors [89]. A study by the Austrian-German Acute Myeloid Leukemia Study Group (AGAMLS) of *FLT3-ITD*+ AML patients who were treated with midostaurin and either relapsed or had refractory response, showed loss of *FLT3-ITD* mutation in nearly half of the patients. In patients who were still positive for *FLT3-ITD*, the clone was different from the dominant diagnostic clone, as demonstrated by mutations in other genes, suggesting that mechanisms other than FLT3 kinase activity contribute to relapse or refractory responses to midostaurin [83].

In more than 50% of cases, no *FLT3* mutation is detected upon the onset of clinical TKI resistance, suggesting that mutations in other genes or pro-survival signals from the bone marrow microenvironment provides alternative survival pathways. Mutations in epigenetic modifiers, the RAS/MAPK pathways *WT1*, and *TP53*, have been observed in resistant clones following FLT3 targeted therapy [89,90,91]. The FLT3 molecule can also be inhibited or inactivated through mechanisms that are alternative to the inhibition of FLT3 kinase activity, and these mechanisms might serve as the basis for future treatment of AML patients [40]. As glycosylation of FLT3 is required for its maturation, inhibition of this process using agents such as Fluvastatin have been shown to inhibit FLT3 activity using in vitro and in vivo models [92]. Inhibiting HSP90, which is required for stabilization of FLT3-ITD, might be another approach, as inhibition of HSP90 has been shown to degrade FLT3-ITD through polyubiquitination and the proteasome machinery [93]. Another approach for suppressing FLT3-ITD and not wild-type FLT3 involves the proteasome pathway through stimulation of the E2 ubiquitin ligase, UBCH8, using the HDAC inhibitor LBH589 [46]. These recent developments in understanding the mechanisms of resistance to FLT3 inhibitors provides the opportunity for developing new potential therapies to improve the outcome of the FLT3-ITD AML patients.

## 4. BCL-2

The B-cell leukemia/lymphoma 2 gene (*BCL-2*) is located on the long arm of chromosome 18, has three exons, and encodes a 26 KD protein consisting of 239 amino acids. The C-terminus of the protein is highly hydrophobic, which leads to its dominant localization within the mitochondrial outer membrane. However, it also localises to the nuclear envelope and the membrane of the endoplasmic reticulum [94]. The BCL-2 protein is expressed by hematopoietic lineages and various other normal tissues such as the epithelium and neurons [95].

BCL-2 belongs to the BCL-2 family of proteins, which is divided into pro-apoptotic and anti-apoptotic families. The pro-apoptotic proteins can be BH3-only proteins or pro-apoptotic multi-domain effector proteins (BAX and BAK). The mechanism of action for the pro-apoptotic family members is through inducing mitochondrial outer membrane permeabilization (MOMP). The BH3-only proteins include BID, BIM, BMF, PUMA, BAD, BIK, HRK, and NOXA [96,97,98]. The BH3 domain of these proteins binds to a hydrophobic groove on anti-apoptotic members of the BCL-2 family to negate their anti-apoptotic action [99]. Binding of the BH3 domain to anti-apoptotic proteins releases BAX and BAK (effectors), which enable them to oligomerise within the mitochondrial outer membrane for MOMP. The other mechanism of apoptosis induction by the BH3-only proteins is to activate BAX and BAK directly [100,101]. The anti-apoptotic protein members include BCL-2, BCL-XL, myeloid cell leukemia sequence 1 (MCL-1), BCL-w, and BFL-1/A1. BCL-2 prevents MOMP by sequestering the pro-apoptotic proteins [102]. This action of BCL-2 and other anti-apoptotic members of this family blocks the release of cytochrome c, a hallmark of mitochondrial apoptosis [103], which consequently prevents apoptosis activation and increases cell survival [104,105]. This anti-apoptotic activity contributes to cancer development and also resistance to chemotherapy, as cancer cells often control the death mechanisms by elevating the expression of BCL-2, BCL-XL, and MCL-1, which neutralises the apoptotic action of the BH3-only proteins of the BCL-2 family [106].

The viability of AML cells depends on BCL-2, and inhibition of BCL-2 results in the death of the AML cells [107]. *BCL-2* has been shown to be overexpressed in CD34^+^ AML cells [108] and associated with poor prognosis and resistance to chemotherapy [109,110]. The higher dependency of AML CD34^+^ cells on BCL-2 has led to therapies based on targeting BCL-2, which might spare normal haematopoietic stem cells (HSC) which were shown to depend more on MCL-1 for their survival [111,112,113]. ‘BH3-mimetic’ inhibitors directly bind to BCL-2 family anti-apoptotic proteins by mimicking the BH3 domain of pro-apoptotic proteins, and as a consequence of this action they antagonise BCL-2 family anti-apoptotic proteins [114]. Venetoclax is a potent and selective inhibitor of BCL-2 and was approved in 2018 by the FDA and later by EMA in combination with either DNA methyltransferase inhibitors (DNMTi’s) or low-dose cytarabine (LDAC) in older or unfit AML patients [115,116]. Venetoclax specifically targets BCL-2 and has a very low affinity to other members of the anti-apoptotic BCL-2 family, like BCL-XL and BCL-W; therefore, this compound does not have any adverse action on platelets [117]. Through a different mechanism, venetoclax in combination with azacytidine has been shown to reduce the leukemia stem cell population in AML by decreasing amino acid uptake and consequently reducing oxidative phosphorylation (OXPHOS), which is essential for LSC survival [118,119,120]. Despite promising responses in various studies, primary and adaptive resistance to venetoclax monotherapy has been observed in AML patients [115]. The success of achieving complete remission with venetoclax monotherapy in relapse or refractory AML was reported to be 19% and in combination therapy between 30–54%; in newly diagnosed patients, nearly 30% do not achieve remission with combination therapy [118,121].

Expression of *BCL-2* is essential for the efficacy of venetoclax, and the expression shift from *BCL-2* to the other anti-apoptotic family members *MCL-1* or *BCL2L1* can cause venetoclax resistance [113,115,122]. The reliance of monocytic AML cells on MCL-1 rather than BCL-2 seems to be the underlying reason for selection of a pre-existing monocytic subpopulation at the time of relapse following ven/aza therapy [123]. The functional genomics screen of the AML cell line model, MOLM13, has shown the inactivation of the *TP53* and *BAX* genes as key elements of venetoclax resistance, explaining venetoclax insensitivity in samples from AML patients who have low expression of these two genes [124]. These data suggest that the mechanism of resistance to venetoclax is through replacement of BCL-2 activity by another anti-apoptotic family member or by inactivation of some major apoptotic genes.

Resistance has also been shown to occur through altering the metabolic target of venetoclax. Untreated LSCs rely on amino acid metabolism as the main source of OXPHOS, and shifting the metabolism toward fatty acid oxidation is considered a mechanism of developing resistance in LSCs at relapse [118]. Mutations of the RAS pathway (*PTPN11*, *KRAS*, *NRAS*) were found to be associated with poor response to ven/aza therapy [118], and this association might be due to their role in enhancing fatty acid metabolism, as demonstrated in lung cancer [125]. The altered metabolism in relapsed AML LSCs compared with de novo AML seems to be responsible for poor or lack of response to ven/aza [126]. The elevated metabolism of nicotinamide in relapsed AML was demonstrated to be responsible for resistance to ven/aza, as it enhances the activity of tricarboxylic acid (TCA) cycle enzymes, including isocitrate dehydrogenase, 2-oxoglutarate dehydrogenase, and malate dehydrogenase, leading to increased metabolism of not only amino acids but also fatty acids. The uptake of nicotinamide in relapsed LSC was demonstrated to be increased and converted to NAD+ by nicotinamide phosphoribosyltransferase (NAMPT), and the inhibition of NAMPT overcame resistance to ven/aza in relapsed LSCs in vitro [126]. A summary of molecular markers associated with response or resistance to venetoclax is summarised in Table 3.

In summary, BCL2 seems to be a novel therapeutic target for AML patients with promising findings through clinical studies. The recent data on the mechanism of venetoclax action highlights the diversity in the pathways through which this drug inhibits AML cells, how metabolic alterations play a role in development of resistance and the requirement for combining venetoclax with other inhibitors to improve the clinical outcome and reduce the rate of relapse.

## 5. IDH1/IDH2

The isoforms 1 and 2 of isocitrate dehydrogenase (*IDH1* and *IDH2* genes), located on chromosomes 2q33 and 16q26, encode for IDH1 and IDH2 catalytic enzymes, respectively [127]. IDHs are a group of homodimeric enzymes involved in cellular metabolism and epigenetic regulation, including adaptation to hypoxia, histone demethylation, and deoxyribonucleic acid (DNA) modification, known to result in altered function in certain tumour cells [128]. IDH1 and IDH2 function as homodimers with two active sites per dimer and each subunit is composed of a large domain, a small domain, and a clasp domain [129]. IDH1 and IDH2 are important components of the TCA cycle, and are responsible for the first of two decarboxylations and dehydrogenations in this process [128]. Under normal physiological conditions, these enzymes catalyse the oxidative decarboxylation of isocitrate to α-ketoglutarate (α-KG) to produce reduced nicotinamide adenine dinucleotide phosphate (NADPH) from NADP+ [128,130,131]. Several dioxygenases rely on this process, as they require sufficient cellular levels of α-KG for metabolic and epigenetic regulation [129]. The evolutionarily conserved arginine residues at Arg132 (IDH1) and Arg172 (IDH2) in the active site of these enzymes are critical for the binding of isocitrate within the catalytic pocket [129].

Somatic mutations in *IDH1* and *IDH2* have been described firstly in glioma and later in AML [127,132]. Together, they are detected in up to 20% of AML patients (6–16% for *IDH1* and 8–19% for *IDH2*), and are enriched in patients with normal karyotypes, with increased prevalence in elderly patients [6,133,134]. *IDH1/2* mutations are early clonal events in disease evolution and occur as heterozygous missense variants with an oncogenic gain of function [128,135]. All *IDH1/2* variants described in AML affect three codons within exon 4 (R132 of *IDH1*, and R140 or R172 of *IDH2*) [128,132]. These mutations have been described to affect the active site of the IDH enzyme, which confer a gain-of-function activity that causes reduction of α-KG to an oncometabolite, the (R) enantiomer of 2-hydroxyglutarate (2-HG) [128,130,131]. Increased levels of 2-HG can competitively inhibit α-KG-dependent dioxygenases, such as the ten eleven translocation (TET) enzyme family and histone lysine demethylases, leading to a ‘hypermethylation signature’ of the downstream target genes, which then leads to a block of myeloid differentiation and the accumulation of immature hematopoietic cells that is characteristic of AML [128,131]. Additionally, altered expression of genes including *PU1*, *RUNX1*, *GATA1*, and *CEBPA* leads to overexpression of the MAPK cell signalling pathway and *HOXA* genes, which together with activation of the extracellular-signal-regulated kinase (ERK)-NF-κB signalling pathway result in impaired differentiation, leukemic cell proliferation, and DNA damage [132].

The prognostic implication of *IDH* mutations in AML is controversial, but seems to be influenced by the specific location of the mutation, other co-occurring mutations, and the risk groups according to genomic profile [128,132,136]. They have been reported to frequently co-occur with *NPM1* and *SRSF2* mutations, while being almost mutually exclusive with both *TET2* and *WT1* mutations [128,130]. Another important aspect of these mutations is the fact that they remain stable during disease progression [132,137,138]. Because they usually occur as early genomic events in disease pathogenesis, they are typically present in dominant clones and persist even after chemotherapy treatment [132]. The prognostic significance of *IDH* mutations in AML is not well known. Long-term persistence of *IDH* mutations using highly sensitive techniques was seen in AML patients who were in molecular remission for *NPM1* mutation following chemotherapy. Their disappearance following allogenic stem cell transplantation concluded their presence in a pre-leukaemia clone and resulted in them not being a suitable marker for monitoring minimal residual disease in AML [139,140].

Over recent years, multiple novel small molecule inhibitors which target mutated *IDH* by binding to the active site of the enzyme and preventing reduction of α-KG to 2-HG have been emerging [128,132,141,142]. In 2013, the first IDH2 inhibitor was developed by Agios Pharmaceuticals. Years later, AG-221 (Enasidenib), an allosteric and non-competitive enzyme inhibitor, was approved for pre-clinical studies [143]. Following the successful preclinical studies of this inhibitor in mouse models, an international, open-label, phase 1/2 trial was launched to investigate the safety and tolerability of enasidenib in patients with relapsed or refractory *IDH2*-mutant AML [143]. These clinical trials were deemed successful, as the overall response rate (ORR) of the drug was 40% when compared with controls [143,144]. IDH1 inhibitors, including AG-120 (Ivosidenib, Agios), a reversible, allosteric and competitive enzyme inhibitor, and IDH305 (Novartis Oncology) also showed evidence of efficacy. Ivosidenib trials were successful for the treatment of *IDH1*-mutated relapsed or refractory AML, with an ORR of 41.6%, and later on also for the treatment of newly diagnosed *IDH1*-mutant AML, with ORR of 54.5% [145,146]. Currently, there are two IDH inhibitors that are FDA-approved for the treatment of AML: enasidenib and ivosidenib. These two agents are not currently approved by EMA. Enasidenib was approved for the treatment of adult patients with relapsed or refractory AML with an *IDH2* mutation in 2017. Ivosidenib was approved by the FDA for patients with relapsed or refractory *IDH1*-mutated AML in 2018, and also as a front-line therapy for newly diagnosed elderly patients 75 years or older or who are ineligible to receive intensive chemotherapy in 2019 [132].

Even though IDH inhibitors have shown promising results, the emergence of resistant subclones has been observed [128]. Co-occurring mutations in the receptor tyrosine kinase (RTK) pathway are associated with both primary and secondary therapeutic resistance to both enasidenib and ivosidenib [147,148]. Baseline mutations in the RTK pathway genes (*NRAS*, *KRAS*, *PTPN11*, *KIT*, *NF1*, *BRAF*, or *FLT3*) were enriched in patients with mutant *IDH1* AML who did not achieve disease remission with ivosidenib treatment [148]. In addition to RTK pathway mutations, a higher mutational burden (≥6 mutations) and/or the presence of a *FLT3* co-mutation were also associated with primary resistance to enasidenib [130,147,148]. Another therapeutic resistance mechanism is the emergence of second-site *IDH* mutations affecting the binding site of the enzyme (i.e., S280F and R119P in IDH1; Q316E and I319M in IDH2) [148,149,150]. The majority of these recently described second-site IDH mutations were associated with a concurrent increase in 2-HG levels that was resistant to targeted therapy [148]. Lastly, secondary resistance was also associated with “isoform switching”, where patients that initially presented with an IDH1 mutation then acquire an IDH2 mutation at the time of relapse, and vice versa [148,151].

Recent studies have revealed new insights into using poly-ADP ribose polymerase (PARP) inhibitors to target *IDH* mutant cells. The accumulation of 2-HG in cells with *IDH* mutations inhibits the function of αKG-dependent dioxygenases (KDM4A and KDM4B) that are critical for the homologous recombination (HR) DNA repair pathway [152,153]. This means that IDH1/2 mutations induce an HR defect and the consequent vulnerability of the tumour cells to PARP inhibition. In vivo studies using mouse models have demonstrated that PARP inhibitors are effective against IDH mutant myeloproferative syndrome (MDS)/AML and can overcome resistance to targeted IDH inhibitors [152]. A proof of concept, biomarker-driven, multi-institution, phase II open label clinical trial is currently investigating the effectiveness of PARP inhibitor monotherapy (olaparib) to treat *IDH* mutant relapsed/refractory AML and MDS [154] (ClinicalTrials.gov Identifier: NCT03953898). The introduction of IDH inhibitors and their promising results from clinical studies along with understanding the mechanisms of resistance to these inhibitors and the exploration of potential agents for overcoming resistance is another example of how target therapy has found its way into management of AML patients for better clinical outcomes.

## 6. Functional Genomics and Target Discovery

The presence of several mutated genes in AML cells and the heterogeneity of these mutations makes the predicted impact of these genomic abnormalities on the activated signalling pathways difficult. The pathologically activated genes may be several steps downstream of the identified driver mutations and are not recognised or easily predicted. An example of the latter is the activation of MKNK1 in *JAK2*-mutated neoplasms [155]. One approach to overcome the complexity of identifying the essential genes or pathways in cancer cells is through inhibiting the function of the genes and assessing the viability of the cancer cells. The genes and signalling pathways whose suppression reduces the viability or proliferation of the cancer cells play major roles in pathogenesis and therefore are potential therapeutic targets. These targets may not necessarily be mutated themselves. Interestingly, some of these genes and signalling pathways may be activated due to the interaction of the cancer cells with the surrounding microenvironment and the signals they received from neighbouring cells [156].

The inhibition of genes or pathways to identify those that are essential for viability or proliferation can be done through chemical inhibition, such as applying a panel of inhibitors targeting different molecules [157,158,159]. For instance, the Beat AML clinical trial was initiated based on the concept of using genomic technology to identify each patient’s cancer-driving genetic mutations, followed by matching patients with the most promising targeted treatment, and the initial observations have been promising [11]. Alternatively, one can use inhibition using small hairpin RNA (shRNA) or CRISPR technology libraries [159,160,161,162]. For the last few years, pooled shRNA libraries have been applied to various cancer cell lines in combination with various drugs to identify the genes that are essential for the viability of the cancer cells, and those whose inhibition demonstrates synthetic lethality with the investigated inhibitor [160,163]. The majority of these studies were performed using cell lines because of their easier manipulation and their in vitro viability. The principle of a pooled shRNA library screen is the transduction of the cancer cells by a pool of various shRNAs targeting a certain number of genes, and culturing the cells for a selection period of 8 to 10 cell divisions. During the selection period, the shRNAs targeting the genes essential for survival of the investigated cancer cells are depleted. The depleted shRNAs are then characterised using next generation sequencing techniques [160,161,163].

This technique and similar methods, such as pooled CRISPR interference (CRISPRi), which similarly works by targeting various genes in a population of cancer cells, provide information on the genes, and consequently pathways, which are essential for cancer cell survival, without necessarily having the knowledge about the underlying genomic abnormalities. These functional genomic techniques measure the outcome of all the interactions between the dysregulated genes (intrinsic) and also the interaction of the cancer cells with their microenvironment (extrinsic), if the screen is performed in the presence of a bone marrow-mimicking microenvironment. Therefore, the generated information is expected to be more comprehensive compared with genomic sequencing alone. However, the validity of the findings from functional genomics depends on the condition under which the screen is performed and how close the experimental condition is to the patients’ bone marrow microenvironment. Although pooled shRNA screens have been used within the last decade by several groups, these studies have been limited to cell line models rather than primary cancer cells. The main challenges on screening primary cells are the low efficiency of the current techniques at transducing primary cells with shRNA or CRISPR vectors, and also the short in vitro survival of the primary cells, which is particularly true for AML [164]. We were able to screen primary AML cells in two independent investigations using a selective pooled shRNA library (selected from reviewing literature on the genes involved in AML pathogenesis and also from high throughput sequencing data) [165], and then a pooled library targeting genes known to be involved in major signalling pathways [161], which identified a few essential genes for the viability in some AML cases. However, further modification to the methodology and larger numbers of AML cases with various subtypes are required before validation of this method as a clinically approved tool for therapeutic target discovery in patients. Development of assays for enrichment and in vitro expansion of leukaemia stem cells (LSC) from patient samples is one of the requirements for improving the pooled shRNA or CRISPRi screening technique. Because of the small number of AML LSCs which can be enriched, and the requirement for a 200-1000-fold incrase of the library complex, a library with the smallest but most relevant shRNAs or guide RNAs (gRNAs) will be required for optimization of the screen. To mimic the bone marrow microenvironment, a standardised three-dimensional culture containing mesenchymal stromal cells and other components of the bone marrow niche [166,167,168] must be developed and optimised to standardise the screen across different samples. CRISPR technology has grown rapidly within the last decade and has become the mainstream technique for functional genomics screens. This technology has revolutionised gene editing, and by providing a faster and more efficient method of gene manipulation, has led to significant progress in understanding the function of genes and how they lead to cancer development [169,170]. Development of new single vector constructs containing both gRNAs and the CRISPR-associated endonuclease (Cas protein, active or inactive) [171] provide an opportunity to screen primary leukaemia cells, as this bypasses the requirement for transducing the cells with two constructs. In summary, new developments in genomics technology such as gene editing/targeting, and cell culture techniques such artificial bone marrow models, are expected to convert the pooled functional genomics screen for target discovery into a routine clinical tool for management of AML patients in the era of personalised medicine.

## 7. Conclusions

The clinical practice of AML is changing mainly due to fast moving genomic technologies which have provided a more detailed picture of the underlying causes of AML, as well as innovations in the design and production of targeted and specific inhibitors. These advances are shifting the treatment of AML from non-specific chemotherapy toward personalized medicine, where AML patients are treated with specific inhibitors based on the specific genomic abnormalities of their leukaemia cells. Development of resistance, however, is a common finding, and identification of new targets to overcome resistance is essential for improving the survival of AML patients. While genomic sequencing provides information on the new acquired mutations at relapse, they may not provide the information on the pathways activated by all the detected mutations. Various functional investigations such as drug screens or functional genomics techniques might provide such information. However, further development in the cell cultures of primary AML cells in a condition mimicking the bone marrow microenvironment will be required before these techniques can be used in clinical practice.

## Figures and Tables

**Table 1:** The frequency of various fusions of *RARA* and sensitivity to targeted therapy [13,14,15,16,17,18].

Fusion	Translocation		Frequency	Sensitivity	
				ATRA	AS_2_O_3_
*PML-RARα*	t(15;17)(q24;q12)	bcr1	~55%	+	+
		bcr2	~5%	+	+
		bcr3	~40%	+	+
		rare variant	rare		
*ZBTB16-RARα* *(also known as PLZF-RARα)*	t(11,17) (q23, q21)		Up to 0.8%	−	−
*NUMA-RARα*	t(11;17) (q13;q21)		rare	+	+
*NPM-RARα*	t(5;17) (q32;q12)		~0.5%	+	+
*BCOR-RARα*	t(X;17) (p11;q21)		rare	+	−
*FIP1L1-RARα*	t(4;17) (q12;q21)		rare	+	NR
*STAT5-RARα*	t(17;17) (q11;q21)		rare	−	−
*PRKAR1A-RARα*	t(17;17) (q12;q21)		rare	+	+

+ sensitive, − resistant, NR not reported.

**Table 2:** The mutated genes at relapse in APL patients treated with ATRA [34,37,39].

Gene	Mutation
*RARA*	Y208N, K227-T233del, K207-Y208del, L224P, K238E, Y208N, R272Q, I273F, R276W, R276Q, T285A, S287L, S287W, G289R, G289E, L290V, G391E, R394W, 412-414del, M413T
*WT1*	R242fs, R462W
*CDK12*	D877N
*KMT2C*	R209W
*KRAS*	G12R, Q61K
*MED12*	R356P, L2162F
*MYB*	I135KfsTer77
*NRAS*	G12R, Q61K
*NSD1*	N1664K, E1948K
*NT5C2*	K404N, R367Q, R246W
*SALL4*	V191M, A149V
*TET2*	K148NfsTer3
*TFE3*	K68T

**Table 3:** Molecular markers associated with development of resistance or prediction of response to venetoclax [115,118,121].

Molecular Marker	Genomic Alterations Associated with Resistance
*FLT3*	*FLT3*-ITD, *FLT3*-TKD(D835H), N676K
*KRAS*	G12D, G13D, Q61H
*NRAS*	G12D, G13R, G12A, Q61H, Q61K, Q61R
*PTPN11*	F71L, A72T
*TP53*	*TP53* loss, R248W, M246K, V272M, A161T, G154V, R342 *, P278H, E336fs
*BAX*	downregulation
*MCL-1*	upregulation
**Molecular Marker**	**Genomic Alterations Associated with Response**
*NPM1*	*NPM1* mutation
*IDH1*	R132C, R132H, R132L, R132Q, R132S
*IDH2*	R140Q, R172G, R172K, R172M, R172S
*SRSF2*	P95L, P95_R102del
*ZRSR2*	K203fs

* Nonsense mutation.
